# Optimization of Basic Emollient Therapy for the Management of Xerosis Cutis

**DOI:** 10.1111/ijd.17791

**Published:** 2025-05-30

**Authors:** Matthias Augustin, Mélanie Brignone

**Affiliations:** ^1^ Institute for Health Services Research in Dermatology and Nursing (IVDP) University Medical Center Hamburg‐Eppendorf (UKE) Hamburg Germany; ^2^ Market Access and Health Economics and Outcomes Research Pierre Fabre Boulogne‐Billancourt France

**Keywords:** adherence, cost‐effectiveness, dry skin, emollient, moisturizer, xerosis cutis

## Abstract

Topical emollients are the mainstay of basic therapy for managing xerosis cutis (dry skin). In general, most emollient formulations are safe and effective for xerosis cutis and diseases associated with dry skin; however, several other factors can vary widely between products, influence patient adherence, and ultimately affect treatment success. This article reviews key practical and clinical considerations when prescribing emollients for xerosis cutis and discusses strategies to optimize treatment and clinical outcomes for these patients. Although the optimal quantity and frequency of emollient use depend on the extent and severity of skin dryness, frequent (i.e., up to several times daily) and liberal application (i.e., up to 600 g/week) are generally recommended. Lipid‐rich ointments are indicated for very dry skin, while hydrophilic creams and lotions are suitable for less severe xerosis cutis; however, treatment decisions should also be guided by other factors (e.g., cosmetic acceptability and practicality) to ensure that patients can adhere to long‐term emollient therapy. Strategies to promote shared decision‐making and improve treatment adherence include patient education, allowing patients to trial a range of emollient products, regularly following‐up with patients, and adjusting treatment to suit their preferences. Emollient therapy is a cost‐effective strategy to manage patients with chronic dry skin conditions; however, access to basic, lower‐cost formulations remains important to minimize the financial burden on patients. Overall, treatment effectiveness and clinical need should be balanced with individual patient preferences to ensure that the full benefits of emollient therapy for xerosis cutis are realized.

## Graphical abstract

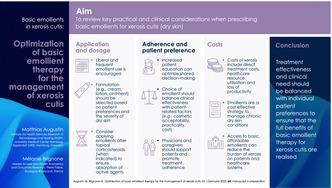

**Summary**

- Why was the study undertaken?
  ○ This article reviews the key practical and clinical considerations when prescribing basic emollients for xerosis cutis and diseases associated with dry skin.
- What does this study add?
  ○ We discuss optimal emollient therapy in terms of formulation, application, and dosage and provide strategies to overcome patient factors that jeopardize long‐term adherence.
- What are the implications of this study for disease understanding and/or clinical care?
  ○ Basic emollients are safe and cost‐effective treatment options for people with xerosis cutis; however, treatment decisions should balance clinical effectiveness with patient preferences to ensure that the full benefits of emollient therapy are realized.

## 1 Introduction

Xerosis cutis is a burdensome dermatological condition characterized by reductions in the water‐holding or barrier functions of the skin [1]. It may be caused by external triggers (e.g., cold weather, low humidity, frequent exposure to water and detergents), endogenous factors (e.g., aging, hormonal changes), or present as a symptom of other diseases, including atopic dermatitis (AD), psoriasis, ichthyosis, and diabetes mellitus [1, 2]. Regardless of its cause, topical emollients can effectively manage xerosis cutis. These vehicle‐type substances typically contain skin‐hydrating humectants (e.g., glycerol, urea, lactic acid) in combination with film‐forming occludents (e.g., liquid paraffin, petrolatum) [1, 2, 3, 4, 5, 6, 7, 8, 9, 10].

A wide range of emollient therapies is available to manage xerosis cutis, which varies in terms of formulation, biophysical properties, level of clinical evidence, cosmetic acceptability, dosage, and cost. Data to describe the comparative efficacy of different emollients are limited; however, it is generally accepted that most emollient formulations are safe and effective strategies to manage dry skin and improve quality of life [11, 12, 13, 14, 15, 16, 17, 18]. Therefore, differentiating and selecting the best emollient therapy for individual patients should be based on additional factors beyond clinical effectiveness. This article aims to review the key considerations when prescribing emollients to patients with xerosis cutis and diseases associated with dry skin to optimize the use and benefits of this therapy.

## 2 Application and Dosage

Clinical guidelines and consensus statements for conditions associated with dry skin consistently advocate regular emollient use for the long‐term management of xerosis cutis [1, 3, 4, 5, 6, 7, 8, 9, 19, 20, 21, 22]. The optimal amount and frequency of emollient use may vary widely based on body surface area and the severity of skin dryness; however, liberal and frequent application is generally recommended [5, 22]. For example, recent European Task Force on Atopic Dermatitis (ETFAD) and EuroGuiDerm guidelines recommend emollient use of ≥ 30 g/day (equivalent to approximately 250 g/week or 1 kg/month) for adults with xerosis cutis associated with AD [5, 19]. On the other hand, best practice guidelines for general emollient use by the British Dermatological Nursing Group (BDNG) recommend quantities of approximately 250 g/week for children and up to 600 g/week for adults, depending on the extent and severity of dry skin [23]. Nevertheless, emollients should be applied as often as needed (i.e., up to several times daily), particularly after bathing, to maximize skin hydration and before bed to minimize overnight water loss [5, 23].

Emollients are available in a range of formulations, including ointments, oils, creams, gels, and lotions. In general, lipid‐rich emollients (e.g., ointments) are recommended for very dry skin due to their occlusive and water‐retaining properties, while hydrophilic and humectant‐containing formulations (e.g., creams and lotions) provide direct hydration for less severe xerosis cutis [1, 23]. However, emollients may ultimately be selected, switched, or used interchangeably based on individual patient preferences [5, 23]. For example, lightweight and fast‐absorbing creams may be favored over greasy ointments for everyday use, but ointments may be more tolerable as an overnight treatment option [23]. Similarly, the choice of emollient formulation may change depending on the season (i.e., summer vs. winter) or body area (e.g., face vs. intertriginous areas) [5].

For inflammatory skin conditions such as AD and psoriasis, emollients are recommended as adjuvants to topical corticosteroids for managing xerosis cutis [5, 6]. However, there is no clear consensus regarding the optimal order or time interval between applying topical emollients and corticosteroids [23, 24]. Although these are likely to depend on the formulation of the emollient (e.g., occlusive products may block corticosteroid absorption), some groups recommend that corticosteroids should be applied to well‐moisturized skin after an emollient has been applied and allowed to absorb [23, 24]. In comparison, UK National Institute for Health and Care Excellence (NICE) guidelines for atopic eczema in children (aged < 12 years) recommend that patients can decide which order to apply emollients and other topical therapies, provided that they are applied one at a time and that “several minutes” have elapsed before applying the second product [25]. Suppose a patient is unable to ensure sufficient absorption time between each product. In that case, it may be prudent to apply the corticosteroid first, thus allowing its active agent to absorb without potential interference. It should also be noted that vehicles used in topical corticosteroids are not inert substances and that the potential therapeutic effects of these excipients should be considered when selecting and applying corticosteroids in conjunction with emollients [26]. Further research is needed to understand better the complex biophysical interactions between topical emollients, corticosteroids, and their respective vehicles.

## 3 Adherence and Patient Preference

Despite the well‐established efficacy and safety of emollients for conditions associated with dry skin, it has been estimated that approximately 40% of patients are non‐adherent to topical dermatological therapies [1, 27]. In patients with chronic inflammatory skin diseases, a systematic review found that treatment adherence rates are lowest for those using topical preparations, greater for oral agents, and highest for subcutaneous or intravenous biological therapies [28]. A multitude of patient‐related, physician‐related, disease‐related, and treatment‐related factors have been found to influence treatment adherence in these patients [1, 28, 29]. In particular, key barriers to topical therapy adherence include poor cosmetic acceptability (e.g., greasiness, odor), impracticality (e.g., time spent to apply, frequency of applications, interference with work or lifestyle), lack of instructions or trust in physician recommendations, perceived lack of effectiveness, concern regarding side effects, and cost [1, 28, 29]. Simply put, if a patient does not like the emollient they have been recommended or believes it will not work, they will not use it.

The success of any treatment is contingent upon good adherence; therefore, involving patients in the decision‐making process may reduce the treatment burden, promote adherence, and improve the effectiveness of emollient therapy for xerosis cutis. However, patients tend to choose lighter and less lipid‐rich emollients than their condition requires, which often leads to a less sustained improvement of skin barrier function. Treatment decisions and adherence may be improved through patient education initiatives (e.g., multidisciplinary training programs, nurse‐led educational sessions, e‐health tools), which aim to exchange knowledge and skills between patients and healthcare providers and empower patients to make informed choices regarding managing their condition [5, 7, 28, 30]. Indeed, randomized clinical trials frequently show that comprehensive patient education programs can improve clinical outcomes and quality of life in patients receiving treatment for chronic inflammatory skin diseases [5, 28, 30, 31, 32, 33, 34, 35].

In addition to increased patient education, other strategies to promote adherence to emollient therapy may include [1, 23, 27, 28, 29, 36]:

- Understanding the patient's skin condition, lifestyle, and treatment preferences, and recommending emollients that meet both their personal and clinical needs.
- Allowing patients to trial a range of suitable products and incorporating their experiences and preferences into treatment decisions.
- Ensuring that treatment regimens are as simple and affordable as possible and providing patients with comprehensive (e.g., verbal and written) instructions on application and dosage.
- Reminding patients about treatment through emails, phone calls, text messages, or smartphone applications.
- Involving family or caregivers during the course of treatment to remind and support the patient outside of the clinic.
- Regularly following‐up with patients to assess adherence and gauge their views on the effectiveness and tolerability of treatment.
- Investigating and understanding reasons for non‐adherence, and working with the patient to adjust their treatment regimen or select a new emollient.

The most effective emollient is one that the patient will use; therefore, increased education and shared decision‐making that balance patient preferences with clinical efficacy should form the basis of treatment for those with dry skin conditions.

## 4 Cost

In addition to the humanistic impact of xerosis cutis on patients and caregivers, it also places a significant financial burden on individuals, societies, and healthcare systems. For example, the annual societal cost of moderate‐to‐severe AD in Europe was recently estimated at €30 billion, which included the indirect costs of reduced work productivity (€15.2 billion), direct costs to healthcare systems (€10.1 billion) and personal costs incurred by patients and their families (€4.7 billion) [37]. Moreover, a survey in Germany evaluated the personal, financial, and time burden associated with managing ichthyosis, and found that most patients reported spending 3–210 min per day applying topical therapies (median, 15 min daily) [38]. In addition to this time burden, most patients reported out‐of‐pocket treatment costs of €3–3480 per quarter (median, €71 per quarter), with ointments accounting for most of this expenditure (median, €50 per quarter) [38].

**FIGURE 1:**
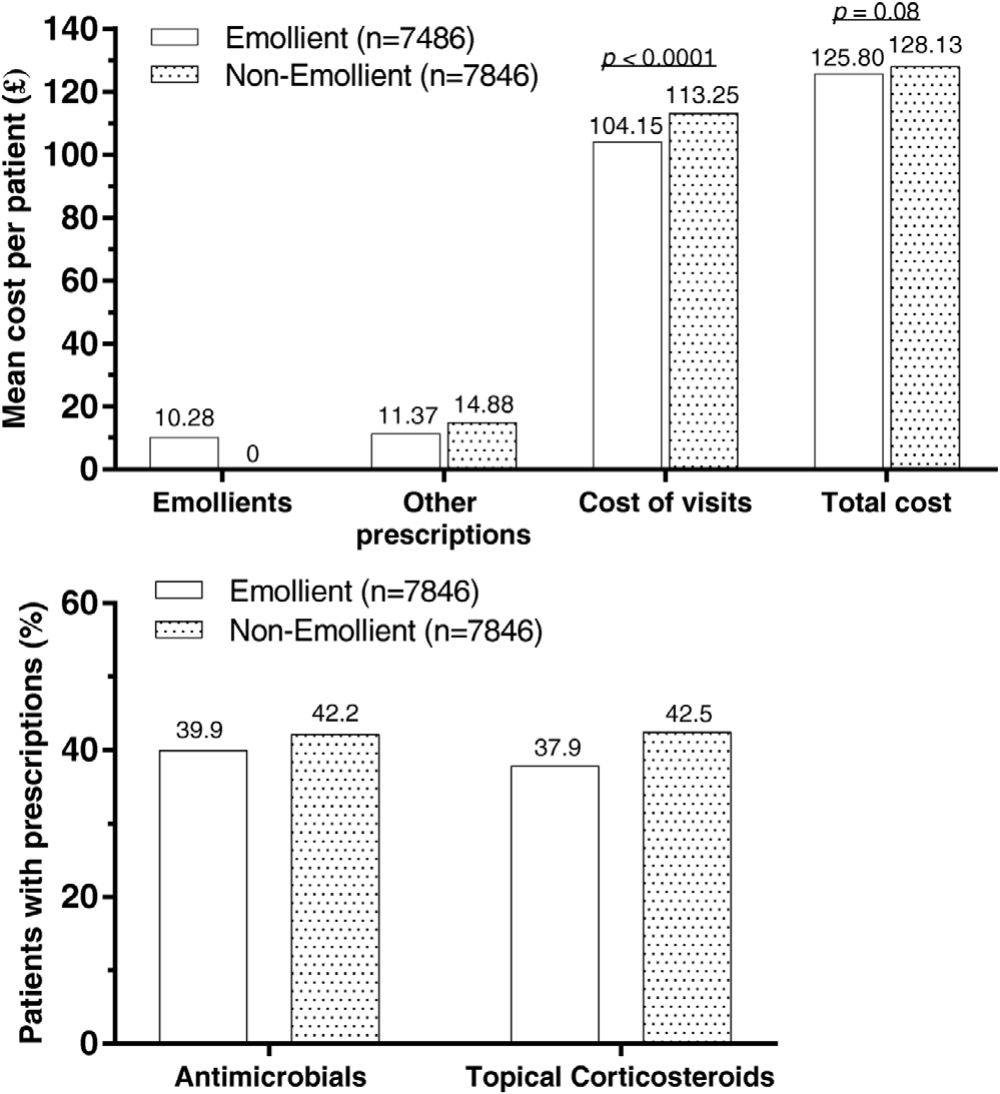
In UK clinical practice, per‐patient healthcare costs (top panel) and medication use (bottom panel) for patients treated with or without emollients for atopic dermatitis [39]. Figure reproduced from Moncrieff, et al. *BMC Dermatol* 2018; 18: 9, under the terms of the Creative Commons Attribution 4.0 International (CC BY 4.0) license (https://creativecommons.org/licenses/by/4.0/).

Indeed, topical emollients account for a large proportion of the personal costs associated with xerosis cutis; however, from a health system perspective, emollient therapy is an overall cost‐effective strategy to manage patients with chronic dry skin conditions. A retrospective analysis of UK primary care data found that prescribing an emollient for patients with AD was associated with significantly fewer primary care visits and prescriptions for potent topical corticosteroids and antimicrobials versus the no‐emollient group [39]. The additional cost of prescribing emollients was offset by the reduced costs of visits and other prescriptions; therefore, total per‐patient costs to the National Health Service were similar between patients treated with and without emollients (Figure 1) [39]. Similarly, a health economic analysis estimated that emollient therapy for AD was cost‐effective versus no treatment in UK clinical practice; this result was driven by improvements in years without flare‐ups, increased quality‐adjusted life years (QALYs), and lower costs associated with reduced healthcare utilization [40]. A subsequent analysis conducted from a French health system perspective also found that emollient therapy was a cost‐effective strategy for managing AD, and was associated with greater time between AD relapses and lower total medical costs versus no emollient [41].

Given the long‐term nature of emollient therapy and the large quantities recommended for optimal dry skin management (approximately 250–600 g/week for adults [5, 23]), out‐of‐pocket costs incurred by patients should nevertheless remain an important factor in clinical decision‐making. The cost of emollients can vary widely depending on brand, formulation, and the addition of active ingredients (referred to as “emollients plus” [42]); however, basic emollients continue to be an effective and accessible treatment option, especially in low‐resource settings. For example, a randomized study of 120 children with AD compared the efficacy of four emollient formulations: cetomacrogol, emulsifying ointment, glycerol/petrolatum, and petroleum jelly [43]. Over 3 months of treatment, improvements in disease severity and quality‐of‐life scores were comparable between the four treatment groups, suggesting that affordable emollients (i.e., glycerol/petrolatum and petroleum jelly) are equivalent to more costly treatments (i.e., cetomacrogol and emulsifying ointment) for the management of AD [43]. Moreover, a health economic analysis estimated that prophylactic use of moisturizers on high‐risk newborns was a cost‐effective strategy to prevent the development of AD, with petrolatum (Vaseline) providing the best cost per QALY of the seven moisturizers tested [44]. Taken together, these data suggest that access to affordable emollient therapies may promote adherence, optimize treatment outcomes, and reduce the socioeconomic burden of dry skin conditions on patients and healthcare systems.

## 5 Conclusions

Topical emollients for xerosis cutis vary widely in terms of formulation, biophysical properties, cosmetic acceptability, dosage, and cost, but overall, they represent safe and cost‐effective strategies to improve clinical outcomes and patient quality of life. The success of emollient therapy for xerosis cutis requires that the optimal formulation be used liberally, frequently, and continuously; therefore, patient education and personalized treatment are important to promote long‐term adherence. The choice of emollient should be a shared decision between patients and physicians that balances effectiveness and clinical need with personal factors, including cosmetic acceptability and cost. Tailoring treatment to suit individual patient preferences is critical to ensure that the full benefits of emollient therapy are realized.

## Conflicts of Interest

M.A. has worked as a lecturer or researcher for the following companies, receiving research grants, consulting fees, and support for travel and meeting attendance: AbbVie, Almirall, Amgen, Beiersdorf, Biogen, Boehringer Ingelheim, Bristol Myers Squibb, Dermira, Eli Lilly, Galderma, GlaxoSmithKline, Hexal, Janssen, LEO, Medac, MSD, Mylan B.V., Novartis, Pfizer, Regeneron, Sandoz, Sanofi‐Genzyme, UCB. M.B. is an employee of Pierre Fabre.
